# Hospital and emergency department discharge against medical advice in Western Australian Aboriginal children aged 0–4 years from 2002 to 2018: A cohort study

**DOI:** 10.1111/ppe.13018

**Published:** 2023-11-20

**Authors:** Daniel Christensen, Alison Gibberd, Bridgette McNamara, Sandra Eades, Carrington Shepherd, David B. Preen, Daniel McAullay, Natalie Strobel

**Affiliations:** ^1^ Centre for Improving Health Services for Aboriginal and Torres Strait Islander Children and Families (ISAC), Kurongkurl Katitjin Edith Cowan University Perth Western Australia Australia; ^2^ Centre for Epidemiology and Biostatistics, Melbourne School of Population and Global Health The University of Melbourne Melbourne Victoria Australia; ^3^ Data Sciences Hunter Medical Research Institute New Lambton Heights New South Wales Australia; ^4^ Barwon South West Public Health Unit Barwon Health Geelong Victoria Australia; ^5^ Curtin Medical School, Faculty of Health Sciences Curtin University Perth Western Australia Australia; ^6^ Ngangk Yira Institute for Change Murdoch University Murdoch Western Australia Australia; ^7^ Telethon Kids Institute The University of Western Australia Nedlands Western Australia Australia; ^8^ School of Population and Global Health The University of Western Australia Crawley Western Australia Australia

**Keywords:** Aboriginal, children, DAMA, data linkage, Western Australia

## Abstract

**Background:**

Discharge against medical advice (DAMA) is a priority issue for the health system. Little is known about the factors associated with DAMA for Aboriginal and/or Torres Strait Islander (Aboriginal) children in Australia.

**Objectives:**

Investigate the associations between DAMA for hospital admissions and emergency department (ED) presentations and: (i) child, family and episode of service characteristics and (ii) 30‐day readmission/ re‐presentation.

**Methods:**

We conducted a cohort study of Aboriginal children born in Western Australia (2002–2013) who had ≥1 hospital admissions (*n* = 16,931) or ED presentations (*n* = 26,546) within the first 5 years of life. The outcome of interest was hospital and ED DAMA and adjusted odds ratio were derived using multilevel mixed‐effects logistic regression.

**Results:**

In the Hospital Cohort, there were 43,149 hospitalisations for 16,931 children, with 684 hospitalisations (1.6%) recorded as DAMA. In the ED Cohort, there were 232,082 ED presentations in 26,546 children, with 10,918 ED presentations (4.7%) recorded as DAMA. DAMA occurring in hospitals between 2014 and 2018, the adjusted odds decreased by 75% compared to the period between 2002 and 2005. The adjusted odds of ED DAMA increased by 46% over the same period. Hospital admissions in regional and remote hospitals were almost seven times the adjusted odds of DAMA compared with hospital admissions in Perth metropolitan hospitals. The adjusted odds of ED DAMA decreased by 12% for ED presentations in regional and remote hospitals compared to those in Perth metropolitan hospitals. There was no evidence of hospital DAMA being associated with hospital readmission within 30 days and limited evidence of ED DAMA being associated with re‐presenting to an ED within 30 days.

**Conclusions:**

The study identified several important determinants of DAMA, including admission status, triage status, location and calendar year. These findings could inform targeted measures to decrease DAMA, particularly in regional and remote communities.

**Synopsis**

**Study question**

This study aimed to examine the associations between DAMA for hospital admissions and ED presentations, and: (i) child, family and episode of service characteristics, as well as (ii) the odds of 30‐day readmission/re‐presentation for Aboriginal children aged <5 years born in Western Australia between 2002 and 2013.

**What is already known**

DAMA is a priority for the health system as it can negatively impact patient well‐being.

**What this study adds**

DAMA is associated with a range of characteristics, including hospital location, mode of admission/ triage code and year of admission/presentation. There was no evidence of hospital DAMA being associated with an increased likelihood of hospital readmission within 30 days and limited evidence of ED DAMA being associated with re‐presenting to an ED within 30 days.

## 1 BACKGROUND

Discharge against medical advice (DAMA) refers to when a patient leaves hospital before discharge is recommended by the treating clinical team.^1^, ^2^ This includes patients admitted to hospital who discharge themselves against medical advice, and patients who leave the emergency department (ED) at their own risk or without waiting to be attended to by a medical officer. DAMA represents an interruption to medical treatment and can result in increased readmission/re‐presentation, morbidity, mortality and cost to the health system.^1^, ^3^, ^4^, ^5^

High rates of DAMA are often seen in populations with concurrent social and health issues, including homelessness, social disadvantage, mental health issues and drug and alcohol use.^5^, ^6^, ^7^, ^8^ Patients with a history of DAMA have a higher likelihood of subsequent DAMA episodes.^5^, ^9^ In the Australian context, Aboriginal and Torres Strait Islander (hereafter respectfully referred to as Aboriginal) people are overrepresented in DAMA episodes.^10^ This discrepancy is more pronounced in the Northern Territory, South Australia and Western Australia and increases with remoteness of residence.^7^, ^10^ In a review of the causes of DAMA, Shaw^3^ highlighted the contribution of health systems with insufficient support for families, cultural safety and culturally appropriate care. As a result, DAMA is an ongoing issue of equity for health systems and is of both national and jurisdictional importance.^10^, ^11^

The focus on DAMA in the scientific literature tends to be on adult populations. However, in Australia, between 2015 and 2017, 1.0% of hospitalisations for Aboriginal children aged <5 years reported a discharge code of left against medical advice/discharged at own risk compared with 0.3% of hospitalisations for non‐Indigenous children.^10^ Likewise, in a study of 124,757 patients, Aboriginal children were 1.6 times more likely to have a DAMA in a tertiary paediatric hospital compared to non‐Aboriginal children.^2^ Predictors of DAMA among children were hospital site, mental health/behavioural diagnosis and emergency rather than elective admission.^2^ For ED presentations between 2015 and 2017, 2.3% of presentations for Indigenous children aged <5 years left at their own risk or did not wait, compared with 1.6% for non‐Indigenous children.^10^ By following a cohort across time, this present study adds to this work and provides a richer use of contextual factors by using linked administrative data.

We investigated the effects of child, family, community and episode of service characteristics on DAMA in Aboriginal children aged <5 years who had one or more hospitalisation or ED presentation. In addition, we investigated whether hospital and ED DAMA was associated with an increased likelihood of 30‐day readmission to hospital or re‐presentation to EDs, respectively.

## 2 METHODS

### 2.1 Cohort selection

The population for this study was based on the ‘Defying the Odds’ cohort and included all Aboriginal children born in Western Australia between 2002 and 2013 (*n* = 29,319).^12^ A child was included in the cohort if they, their parents or grandparents identified as Aboriginal using an algorithm applied to the Aboriginal status indicators within the multiple data sets by the Western Australia Data Linkage Branch.^13^ We excluded children if their full siblings were not identified as Aboriginal. The study cohort's relatives were identified by Western Australia's records of family links, the Family Connections System.^14^ This study used two cohorts derived from the Defying the Odds Cohort: (i) the Hospital Cohort (Figure S1), which included children aged <5 years with at least one hospitalisation (57.7%; 16,931/29,319) and (ii) the Emergency Department Cohort (Figure S2), which included children aged <5 years with at least one ED presentation (90.5%; 26,546/29,319). These cohorts were defined and analysed separately.

The follow‐up period for each child in the study was from birth up until their fifth birthday. Children who died prior to 5 years of age during the study period (excluding stillbirths) were retained in the analysis cohorts (Hospital Cohort, *n* = 77; Emergency Department Cohort, *n* = 144), as they were eligible for hospital admissions and ED presentations up to the time of their death.

### 2.2 Data source

Linked data from the Midwives Notification System (MNS), the Emergency Department Data Collection (EDDC), Death Registrations and the Hospital Morbidity Data Collection (HMDC) were used. Probabilistic linkage of all records, by matching identifiers (e.g. name, address, date of birth, etc) across sets of records, was undertaken by the Western Australia Data Linkage Branch from the Western Australia Department of Health to produce deidentified records for analysis.^15^ Audits of data linkage quality have shown a high degree of accuracy.^16^, ^17^

The MNS includes clinical (infant weight, gestational age, parity) and socio‐demographic (mother's age, socio‐economic status, remoteness) data on all Western Australian live births and stillbirths of more than 20 weeks gestation or birthweight >400 g, which are reported by trained midwives within 48 h of delivery. The HMDC and EDDC include data on all completed hospital admissions and ED presentations to all public and private hospitals in Western Australia, respectively.^18^, ^19^ These data are entered by trained medical records staff following the episode of service.

### 2.3 Exposures

#### 2.3.1 Child and maternal characteristics

Characteristics of interest were sex, plurality (one birth from the pregnancy or two or more births), gestational age (<37 or ≥37 weeks), birthweight (<2500 and ≥2500 g), Appearance, Pulse, Grimace, Activity and Respiration at 5 min (APGAR 5) score (<7 or ≥7), parity (<3 or ≥3 previous births) and maternal age at birth (<20 years old or ≥ 20 years old).

#### 2.3.2 Community characteristics

Area level Index of Relative Socio‐Economic Disadvantage and the Accessibility/Remoteness Index of Australia (ARIA) remoteness index, both from the 2016 Australian Bureau of Statistics' Census of Population and Housing, were based on the mother's address at the birth of the child. Area level disadvantage was categorised as state quintiles from most disadvantaged (1) to least disadvantaged (5). Remoteness classifies geographic location on the basis of isolation and distance from service centres and healthcare facilities. These data were split into four categories from least remote (major cities) to most remote (very remote areas).

#### 2.3.3 Episode of service characteristics

The child's age at each episode of service was recorded, and we classified children as infants (<1 year old) or older (≥1 to <5). The calendar year of the episode of service was also recorded.

Any previous DAMA was defined based on whether the child had any history of DAMA, prior to the current episode of service, using the definitions of DAMA given in the Outcomes section. Any previous DAMA was defined separately for hospitalisation admissions and ED presentations. Potentially preventable hospitalisations for children were defined based on the ICD‐10‐AM code from the patient's principal diagnosis, using the scheme defined in Falster et al.^20^ The hospitalisations data contained five classifications of admission status: emergency admissions, elective – waitlist, elective – not waitlist, emergency – emergency department admission and emergency – direct admission.^21^ We combined the three emergency admission classifications into a single category to address changes in coding in older records. The triage code for ED presentations is graded into five categories, based on the Australasian Triage Scale,^22^ from most (resuscitation: immediate) to least urgent (non‐urgent: within 120 min). Supplementary triage codes (dead on arrival, direct admission, inpatient) were excluded due to small cell sizes. Hospital location was classified as either metropolitan or regional and remote based on the WA Department of Health districts; all hospitals outside the Greater Perth metropolitan area were classified as regional and remote.

### 2.4 Outcomes

Hospital admission was defined as any admission to a Western Australian hospital ward for care, including all neonatal nurseries. It excluded the normal postnatal hospital stay for healthy babies. Hospital admissions that were serial transfers (patient moved between hospitals successively without returning home), nested transfers (patient moves to another hospital during an admission) or overlapping transfers (admission date prior to separation date on previous record) were considered a single event.^23^ An ED presentation was defined as any presentation to the ED regardless of whether the child was admitted to hospital. Each hospital admission and ED presentation was considered a single episode of service.

A hospital separation was defined as DAMA if the mode of separation for that admission was recorded in the HMDC as ‘left against medical advice/discharge at own risk’.^19^ An ED discharge was defined as having a DAMA if the discharge status for that presentation was recorded in the EDDC as either ‘did not wait to be attended by a medical officer’ or ‘left at own risk’.^18^ These are headline indicators for the WA Department of Health, and there is a comprehensive recording system to capture these data. For example, emergency patients are given three attempts to respond, and hospital admission records can be corrected if a patient is later found.^1^

30‐day readmission/ re‐presentation is a standard metric for hospitals.^24^ A child was considered to be readmitted to hospital within 30 days if the admission date was within 30 days of the separation date of their previous hospital admission. A child was considered to be re‐presenting to an ED within 30 days if the presentation date was within 30 days of the discharge date of their previous ED presentation.

### 2.5 Statistical analyses

For all exposures we estimated adjusted odds ratios using multilevel mixed‐effects logistic regression, which took into account the nested data structure (children recording multiple episodes of service and children sharing the same mother within their respective study cohort), using a three‐level analysis (observations, children, mothers).

Directed acyclic graphs (DAGs) were used to assess potentially confounding causal relationships for the characteristics of interest (Figures S3 and S4).^25^, ^26^ Based on the DAGs, a minimal sufficient adjustment set (MSA) to deconfound each exposure was identified in DAGitty 3.0.^27^ The adjusted odds ratios presented in this paper should be interpreted as conditional upon the nested data structure in the mixed‐effects models, as well as upon adjustment for the MSA for that variable.

Statistical adjustment was based on conditioning in *melogit* using Stata V.16.0.^28^

### 2.6 Missing data

No imputation was undertaken and missing data were excluded from all analyses, as missing data were negligible (<1%), with missing cell counts specified within all the tables. Data were missing due to either the non‐completion of administrative data or (in the case of area disadvantage or remoteness data) address data which could not be coded. Small cell counts (*n* ≤ 5) have been suppressed for confidentiality.

## 3 RESULTS

### 3.1 Episodes of DAMA hospitalisations

In the Hospital Cohort, there were a total of 43,149 hospitalisations for 16,931 children (Table 1), with 684 hospitalisations (1.6%) recorded as DAMA (Table 2). Children with a birthweight of <2500 g had an adjusted odds ratio for DAMA 30% greater than children with a birthweight of ≥2500 g (Table 2). The adjusted odds of hospitalisation DAMA increased with remoteness from the Perth metropolitan area: compared with children born in major cities of Australia, children born in regional Australia had an aOR of 2.08 (95% CI 1.53, 2.82), children born in remote Australia had an aOR of 3.45 (95% CI 2.55, 4.66), and children born in very remote Australia had an aOR of 4.69 (95% CI 3.64, 6.06).

**TABLE 1:** Socio‐demographic characteristics of the study population, 2002–2013.

Characteristic	Hospital cohort			Emergency Department cohort		
	Total children (*n* = 16,931)	Children with no DAMA (*n* = 16,326)	Children with ≥1 DAMA (*n* = 605)	Total children (*n* = 26,564)	Children with no DAMA (*n* = 19,190)	Children with ≥1 DAMA (*n* = 7374)
Child						
Sex						
Female	7780 (46.0%)	7520 (46.1%)	260 (43.0%)	12,962 (48.8%)	9420 (49.1%)	3542 (48%)
Male	9151 (54.0%)	8806 (53.9%)	345 (57.0%)	13,602 (51.2%)	9770 (50.9%)	3832 (52%)
Plural birth						
Yes	471 (2.8%)	454 (2.8%)	17 (2.8%)	663 (2.5%)	516 (2.7%)	147 (2%)
No	16,460 (97.2%)	15,872 (97.2%)	588 (97.2%)	25,901 (97.5%)	18,674 (97.3%)	7227 (98%)
Gestational age at birth						
<37 weeks	2541 (15.0%)	2418 (14.8%)	123 (20.3%)	3373 (12.7%)	2372 (12.4%)	1001 (13.6%)
≥37 or more weeks	14,390 (85.0%)	13,908 (85.2%)	482 (79.7%)	23,191 (87.3%)	16,818 (87.6%)	6373 (86.4%)
Birthweight						
<2500 g	2369 (14.0%)	2234 (13.7%)	135 (22.3%)	3167 (11.9%)	2223 (11.6%)	944 (12.8%)
≥2500 g	14,562 (86.0%)	14,092 (86.3%)	470 (77.7%)	23,397 (88.1%)	16,967 (88.4%)	6430 (87.2%)
APGAR 5						
<7	370 (2.2%)	359 (2.2%)	11 (1.8%)	496 (1.9%)	348 (1.8%)	148 (2%)
≥7	16,520 (97.6%)	15,928 (97.6%)	592 (97.9%)	26,015 (97.9%)	18,797 (98%)	7218 (97.9%)
Missing	97 (0.6%)	95 (0.6%)	2 (0.3%)	416 (1.6%)	405 (2.1%)	11 (0.1%)
Mother						
Parity						
≥3 previous births	4339 (25.6%)	4162 (25.5%)	177 (29.3%)	6567 (24.7%)	4947 (25.8%)	1620 (22%)
<3 previous births	12,592 (74.4%)	12,164 (74.5%)	428 (70.7%)	19,997 (75.3%)	14,243 (74.2%)	5754 (78%)
Maternal age at birth						
<20 years old	3718 (22.0%)	3565 (21.8%)	153 (25.3%)	5611 (21.1%)	3772 (19.7%)	1839 (24.9%)
≥20 years old	13,213 (78.0%)	12,761 (78.2%)	452 (74.7%)	20,953 (78.9%)	15,418 (80.3%)	5535 (75.1%)
Community						
Area socio‐economic index						
1 (most disadvantaged)	3739 (22.1%)	3540 (21.7%)	199 (32.9%)	5363 (20.2%)	3801 (19.8%)	1562 (21.2%)
2	3271 (19.3%)	3179 (19.5%)	92 (15.2%)	5280 (19.9%)	3767 (19.6%)	1513 (20.5%)
3	3328 (19.7%)	3222 (19.7%)	106 (17.5%)	5292 (19.9%)	3844 (20%)	1448 (19.6%)
4	3348 (19.8%)	3236 (19.8%)	112 (18.5%)	5344 (20.1%)	3886 (20.3%)	1458 (19.8%)
5 (least disadvantaged)	3224 (19.0%)	3130 (19.2%)	94 (15.5%)	5260 (19.8%)	3871 (20.2%)	1389 (18.8%)
Missing	49 (0.3%)	47 (0.3%)	2 (0.3%)	157 (0.6%)	152 (0.8%)	5 (0.1%)
Remoteness area						
Major cities of Australia	6654 (39.3%)	6569 (40.2%)	85 (14.0%)	11,015 (41.5%)	8455 (44.1%)	2560 (34.7%)
Regional Australia	3615 (21.4%)	3517 (21.5%)	98 (16.2%)	5987 (22.5%)	4198 (21.9%)	1789 (24.3%)
Remote Australia	2406 (14.2%)	2303 (14.1%)	103 (17.0%)	3894 (14.7%)	2493 (13%)	1401 (19%)
Very remote Australia	4235 (25.0%)	3918 (24.0%)	317 (52.4%)	5643 (21.2%)	4023 (21%)	1620 (22%)
Missing	49 (0.3%)	47 (0.3%)	2 (0.3%)	157 (0.6%)	152 (0.8%)	5 (0.1%)

**TABLE 2:** Associations between characteristics and hospitalisation DAMA, 2002–2018.

Characteristics	All hospitalisations (*n* = 43,149)	No DAMA (*n* = 42,465)	≥1 DAMA (*n* = 684)	OR (95% CI)	aOR (95% CI)	Minimal sufficient adjustment set used in adjusted models (see Figure S3)
Child						
Sex						
Female	18,799	18,496 (98.4%)	303 (1.6%)	1.00 (reference)	1.00 (reference)	No adjustment is necessary to estimate the total effect of Sex on DAMA
Male	24,350	23,969 (98.4%)	381 (1.6%)	1.00 (0.84, 1.19)	1.00 (0.84, 1.19)	
Plural birth						
Yes	1316	1296 (98.5%)	20 (1.5%)	0.89 (0.52, 1.53)	0.91 (0.53, 1.56)	Maternal age at birth
No	41,833	41,169 (98.4%)	664 (1.6%)	1.00 (reference)	1.00 (reference)	
Gestational age at birth						
<37 weeks	8417	8267 (98.2%)	150 (1.8%)	1.18 (0.95, 1.46)	1.19 (0.95, 1.48)	Area socio‐economic index, Maternal age at birth, Plural birth, Remoteness Area
≥37 weeks	34,732	34,198 (98.5%)	534 (1.5%)	1.00 (reference)	1.00 (reference)	
Birthweight						
<2500 g	8034	7875 (98.0%)	159 (2.0%)	1.39 (1.12, 1.72)	1.31 (1.00, 1.72)	Area socio‐economic index, Gestational age at birth, Maternal age at birth, Parity, Plural birth, Remoteness Area, Sex, Year of Admission
≥2500 g	35,115	34,590 (98.5%)	525 (1.5%)	1.00 (reference)	1.00 (reference)	
APGAR 5						
<7	1373	1359 (99.0%)	14 (1.0%)	0.62 (0.34, 1.14)	0.54 (0.29, 1.01)	Birthweight, Gestational age at birth, Plural birth
≥7	41,679	41,011 (98.4%)	668 (1.6%)	1.00 (reference)	1.00 (reference)	
Missing	97	95 (97.9%)	2 (2.1%)			
Mother						
Parity						
≥3 previous births	11,915	11,711 (98.3%)	204 (1.7%)	1.07 (0.88, 1.29)	1.01 (0.83, 1.24)	Area socio‐economic index, Maternal age at birth, Remoteness Area, Year of Admission
<3 previous births	31,234	30,754 (98.5%)	480 (1.5%)	1.00 (reference)	1.00 (reference)	
Maternal age at birth						
<20 years old	9522	9357 (98.3%)	165 (1.7%)	1.21 (0.99, 1.48)	1.13 (0.93, 1.38)	Area socio‐economic index, Remoteness Area, Year of Admission
≥20 years old	33,627	33,108 (98.5%)	519 (1.5%)	1.00 (reference)	1.00 (reference)	
Community						
Area socio‐economic index						
1 (most disadvantaged)	10,529	10,299 (97.8%)	230 (2.2%)	1.60 (1.22, 2.09)	0.98 (0.74, 1.30)	Potentially preventable hospitalisation, Remoteness Area, Sex, Year of Admission
2	8020	7920 (98.8%)	100 (1.2%)	0.94 (0.69, 1.28)	0.93 (0.68, 1.27)	
3	8371	8260 (98.7%)	111 (1.3%)	1.01 (0.74, 1.36)	0.94 (0.69, 1.28)	
4	8436	8304 (98.4%)	132 (1.6%)	1.11 (0.83, 1.49)	1.05 (0.78, 1.41)	
5 (least disadvantaged)	7744	7635 (98.6%)	109 (1.4%)	1.00 (reference)	1.00 (reference)	
Missing	49	47 (95.5%)	2 (4.1%)			
Remoteness area						
Major cities of Australia	15,038	14,952 (99.4%)	86 (0.6%)	1.00 (reference)	1.00 (reference)	Year of Admission
Regional Australia	8581	8474 (98.8%)	107 (1.2%)	2.17 (1.60, 2.94)	2.08 (1.53, 2.82)	
Remote Australia	6033	5909 (97.9%)	124 (2.1%)	3.51 (2.60, 4.75)	3.45 (2.55, 4.66)	
Very remote Australia	13,448	13,083 (97.3%)	365 (2.7%)	4.91 (3.80, 6.35)	4.69 (3.64, 6.06)	
Missing	49	47 (95.9%)	2 (4.1%)			
Episode of service						
Any previous Hospitalisation DAMA						
Yes	1443	1364 (94.5%)	79 (5.5%)	1.32 (0.90, 1.92)	0.75 (0.43, 1.30)	APGAR 5, Area socio‐economic index, Birthweight, Gestational age at birth, Maternal age at birth, Potentially preventable hospitalisation, Parity, Remoteness Area, Sex, Year of Admission
No	41,706	41,101 (98.5%)	605 (1.5%)	1.00 (reference)	1.00 (reference)	
Age at admission (years)						
Infant (<1)	15,969	15,671 (98.1%)	298 (1.9%)	1.27 (1.08, 1.49)	1.24 (1.05, 1.45)	APGAR 5, Area socio‐economic index, Remoteness Area
Other (1 < 5)	27,180	26,794 (98.6%)	386 (1.4%)	1.00 (reference)	1.00 (reference)	
Year of admission (years)						
2002–2005	8409	8170 (97.2%)	239 (2.8%)	1.00 (reference)	1.00 (reference)	No adjustment is necessary to estimate the total effect of Year of admission on DAMA
2006–2009	13,162	12,962 (98.5%)	200 (1.5%)	0.52 (0.42, 0.64)	0.52 (0.42, 0.64)	
2010–2013	14,959	14,762 (98.7%)	197 (1.3%)	0.46 (0.37, 0.57)	0.46 (0.37, 0.57)	
2014–2018	6619	6571 (99.3%)	48 (0.7%)	0.25 (0.18, 0.34)	0.25 (0.18, 0.34)	
Potentially preventable hospitalisation						
Yes	23,657	23,268 (98.4%)	389 (1.6%)	1.01 (0.86, 1.19)	0.91 (0.78, 1.07)	Area socio‐economic index, Remoteness Area, Sex, Year of Admission
No	19,492	19,197 (98.5%)	295 (1.5%)	1.00 (reference)	1.00 (reference)	
Admission status						
Elective from waitlist	6217	6209 (99.9%)	8 (0.1%)	1.00 (ref)	1.00 (ref)	Area socio‐economic index, Hospital location, Parity, Remoteness Area, Year of admission, Age at admission
Elective not from waitlist	3737	3699 (99%)	38 (1%)	7.72 (3.56, 16.73)	3.42 (1.56, 7.47)	
Emergency admission	33,195	32,557 (98.1%)	638 (1.9%)	14.25 (7.05, 28.82)	6.18 (3.03, 12.59)	
Hospital location						
Perth metropolitan area	18,693	18,630 (99.7%)	63 (0.3%)	1.00 (reference)	1.00 (reference)	Remoteness Area, Year of Admission
Regional and remote	24,456	23,835 (97.5%)	621 (2.5%)	7.70 (5.88, 10.08)	6.99 (4.98, 9.80)	

Children with a prior history of hospitalisation DAMA had an adjusted odds ratio for DAMA 25% less than children without any previous history of hospitalisation DAMA. Children aged <1 at hospital admission had an adjusted odds ratio for DAMA 24% greater than children aged ≥1 to <5 years at admission. The adjusted odds of hospitalisation DAMA decreased over time: compared with admissions in the period 2002–2005, the aOR for DAMA was 0.52 (95% CI 0.42, 0.64) in 2006–2009, 0.46 (95% CI 0.37, 0.57) in 2010–2013 and 0.25 (95% CI 0.18, 0.34) in 2014–2018. There were substantial differences in the adjusted odds of DAMA, based on admission status: children with admission statuses of ‘elective not from waitlist’ (aOR 3.42, 95% CI 1.56, 7.47), and ‘emergency admission’ (aOR 6.18, 95% CI 3.03, 12.59) had increased adjusted odds of DAMA compared with children who were admitted as ‘elective from waitlist’. Children who attended a hospital outside the Perth metropolitan area had nearly 7 times the adjusted odds of DAMA compared with children who attended in the Perth metropolitan area.

There was no evidence of an effect for the remaining exposures on DAMA hospitalisations (Table 2).

### 3.2 Episodes of DAMA emergency department presentations

In the Emergency Department Cohort, there were a total of 232,082 ED presentations in 26,546 children (Table 1), with 10,918 ED presentations (4.7%) recorded as DAMA (Table 3). ED DAMA comprised the discharge codes of ‘did not wait’ and ‘left at own risk’, with ‘did not wait’ contributing 96% of all ED DAMAs (Figure S5).

**TABLE 3:** Associations between characteristics and emergency department DAMA, 2002–2018.

Characteristics	All presentations (*n* = 232,082)	No DAMA (*n* = 221,164)	≥1 DAMA (*n* = 10,918)	OR (95% CI)	aOR (95% CI)	Minimal sufficient adjustment set used in adjusted models (see Figure S4)
Child						
Sex						
Female	107,990	102,780 (95.2%)	5210 (4.8%)	1.00 (reference)	1.00 (reference)	No adjustment is necessary to estimate the total effect of Sex on DAMA.
Male	124,092	118,384 (95.4%)	5708 (4.6%)	0.96 (0.91, 1.00)	0.96 (0.91, 1.00)	
Plural birth						
Yes	5238	5033 (96.1%)	205 (3.9%)	0.79 (0.66, 0.94)	0.80 (0.67, 0.95)	Maternal age at birth
No	226,844	216,131 (95.3%)	10,713 (4.7%)	1.00 (reference)	1.00 (reference)	
Gestational age at birth						
<37 weeks old	33,718	32,246 (95.6%)	1472 (4.4%)	0.93 (0.86, 0.99)	0.95 (0.89, 1.02)	Area socio‐economic index, Maternal age at birth, Plural birth, Remoteness Area
≥37 or more weeks	198,364	188,918 (95.2%)	9446 (4.8%)	1.00 (reference)	1.00 (reference)	
Birthweight						Area socio‐economic index, Gestational age at birth, Maternal age at birth, Parity, Plural birth, Remoteness Area, Sex, Year of Presentation
<2500 g	30,980	29,608 (95.6%)	1372 (4.4%)	0.93 (0.87, 1.00)	0.99 (0.91, 1.08)	
≥2500 g	201,102	191,556 (95.3%)	9546 (4.7%)	1.00 (reference)	1.00 (reference)	
APGAR 5						
<7	5140	4915 (95.6%)	225 (4.4%)	0.95 (0.80, 1.12)	0.97 (0.82, 1.15)	Birthweight, Gestational age at birth, Plural birth
≥7	226,526	215,844 (95.3%)	10,682 (4.7%)	1.00 (reference)	1.00 (reference)	
Missing	416	405 (97.4%)	11 (2.6%)			
Mother						
Parity						
≥3 previous births	57,264	54,843 (95.8%)	2421 (4.2%)	0.87 (0.83, 0.93)	0.92 (0.87, 0.97)	Area socio‐economic index, Maternal age at birth, Remoteness Area, Year of Presentation
<3 previous births	174,818	166,321 (95.1%)	8497 (4.9%)	1.00 (reference)	1.00 (reference)	
Maternal age at birth						
<20 years old	54,972	52,145 (94.9%)	2827 (5.1%)	1.12 (1.06, 1.18)	1.14 (1.08, 1.21)	Area socio‐economic index, Remoteness Area, Year of Presentation
≥20 years old	177,110	169,019 (95.4%)	8091 (4.6%)	1.00 (reference)	1.00 (reference)	
Community						
Area socio‐economic index						
1 (most disadvantaged)	60,440	58,040 (96%)	2400 (4%)	0.78 (0.72, 0.84)	0.93 (0.86, 1.01)	Remoteness Area, Year of Presentation
2	45,888	43,707 (95.2%)	2181 (4.8%)	0.94 (0.87, 1.02)	0.99 (0.91, 1.07)	
3	43,747	41,573 (95%)	2174 (5%)	0.95 (0.88, 1.03)	1.02 (0.95, 1.11)	
4	44,495	42,292 (95%)	2203 (5%)	0.94 (0.87, 1.02)	1.00 (0.93, 1.08)	
5 (least disadvantaged)	37,355	35,400 (94.8%)	1955 (5.2%)	1.00 (reference)	1.00 (reference)	
Missing	157	152 (96.8%)	5 (3.2%)			
Remoteness area						
Major cities of Australia	65,405	61,914 (94.7%)	3491 (5.3%)	1.00 (reference)	1.00 (reference)	Year of Presentation
Regional Australia	50,257	47,536 (94.6%)	2721 (5.4%)	1.03 (0.96, 1.10)	1.03 (0.96, 1.10)	
Remote Australia	40,040	37,814 (94.4%)	2226 (5.6%)	1.03 (0.96, 1.11)	1.03 (0.96, 1.11)	
Very remote Australia	76,223	73,748 (96.8%)	2475 (3.2%)	0.60 (0.56, 0.65)	0.61 (0.57, 0.65)	
Missing	157	152 (96.8%)	5 (3.2%)			
Episode of service						
Any previous Emergency DAMA						
Yes	50,001	46,457 (92.9%)	3544 (7.1%)	1.23 (1.17, 1.30)	1.24 (1.18, 1.31)	APGAR 5, Area socio‐economic index, Birthweight, Gestational age at birth, Maternal age at birth, Parity, Remoteness Area, Sex, Year of Presentation
No	182,081	17,4707 (96%)	7374 (4%)	1.00 (reference)	1.00 (reference)	
Age at presentation (years)						
Infant (<1)	73,152	70,282 (96.1%)	2870 (3.9%)	0.74 (0.71, 0.78)	0.74 (0.71, 0.78)	APGAR 5, Area socio‐economic index, Remoteness Area
Other (1 < 5)	158,930	150,882 (94.9%)	8048 (5.1%)	1.00 (reference)	1.00 (reference)	
Year of presentation (years)						
2002–2005	36,567	35,252 (96.4%)	1315 (3.6%)	1.00 (reference)	1.00 (reference)	No adjustment is necessary to estimate the total effect of Year of presentation on DAMA
2006–2009	76,692	72,937 (95.1%)	3755 (4.9%)	1.39 (1.30, 1.49)	1.39 (1.30, 1.49)	
2010–2013	80,261	76,408 (95.2%)	3853 (4.8%)	1.34 (1.25, 1.44)	1.34 (1.25, 1.44)	
2014–2018	38,562	36,567 (94.8%)	1995 (5.2%)	1.46 (1.35, 1.58)	1.46 (1.35, 1.58)	
Triage code						
Resuscitation: immediate	797	—^a^	—^a^	0.55 (0.17, 1.77)	0.56 (0.17, 1.83)	Area socio‐economic index, Hospital location, Parity, Remoteness Area, Year of presentation, Age at presentation
Emergency: within 10 min	6665	—^a^	—^a^	1.00 (ref)	1.00 (ref)	
Urgent: within 30 min	51,655	50,421 (97.6%)	1234 (2.4%)	3.58 (2.66, 4.82)	3.72 (2.76, 5.00)	
Semi‐urgent: within 60 min	120,810	113,660 (94.1%)	7150 (5.9%)	9.88 (7.37, 13.24)	10.36 (7.73, 13.90)	
Non‐urgent: within 120 min	52,081	49,603 (95.2%)	2478 (4.8%)	9.44 (7.02, 12.69)	11.49 (8.54, 15.45)	
Missing	74	67 (90.5%)	7 (9.5%)			
Hospital location						
Perth metropolitan area	60,355	57,041 (94.5%)	3314 (5.5%)	1.00 (ref)	1.00 (ref)	Remoteness Area, Year of Presentation
Regional and remote	171,727	164,123 (95.6%)	7604 (4.4%)	0.82 (0.78, 0.87)	0.88 (0.82, 0.94)	

^a^ Cells suppressed due to small cell size.

Children who were born in a plural birth had adjusted odds of ED DAMA 20% less than singletons (Table 3). Children with mothers with ≥3 previous births had a 8% decrease in adjusted odds of having a ED DAMA compared with children of mothers with <3 previous births. Younger mothers (aged <20 years old) had a 13% increase in the adjusted odds of having a child who had an ED DAMA compared to older mothers (aged ≥20 years old at the birth of the study child). There was not a consistent pattern between remoteness at birth and the adjusted odds of ED DAMA: compared with children born in major cities of Australia, children born in regional Australia had an aOR of 1.03 (95% CI 0.96, 1.10), children born in remote Australia had an aOR of 1.03 (95% CI 0.96, 1.11), and children born in very remote Australia had an aOR of 0.61 (95% CI 0.57, 0.65).

Compared with children without any previous history of ED DAMA, children with a prior history of ED DAMA were at 24% increased adjusted odds of ED DAMA. Compared with children aged ≥1 to <5 years at ED presentation, children aged <1 at ED presentation were at 26% decreased adjusted odds of ED DAMA. There was a general trend for ED DAMA to increase over time: compared with ED presentations in the period 2002–2005, the aOR for DAMA was 1.39 (95% CI 1.30, 1.49) in 2006–2009, 1.34 (95%CI 1.25, 1.44) in 2010–2013 and 1.46 (95% CI 1.35, 1.58) in 2014–2018. There was a strong pattern of increased adjusted odds of ED DAMA with decreased urgency in triage code: compared with the triage code of emergency, the triage codes of urgent (aOR 3.72, 95% CI 2.76, 5.00), semi‐urgent (aOR 10.36 95% CI 7.73, 13.90) and non‐urgent (aOR 11.49, 95% CI 8.54, 15.45) were all associated with increased adjusted odds of DAMA. The triage code of resuscitation was associated with decreased adjusted odds of DAMA (aOR 0.56, 95% CI 0.17, 1.83). Children who presented to an ED outside the Perth metropolitan area had 12% decreased adjusted odds of DAMA compared with children who attended in the Perth metropolitan area.

There was no evidence of an effect for the remaining exposures on ED DAMA (Table 3).

### 3.3 A comparison of characteristics for metropolitan and regional and remote hospitalisations and emergency department presentations

Given the large differences in the odds of DAMA between metropolitan and regional and remote hospitals, particularly for hospitalisations, an exploratory comparison of the association between the predictive characteristics used in our analyses and hospital location is provided in Tables S1 and S2. These analyses have been conducted separately for hospital admissions and ED presentations. These analyses were limited by small cell counts, particularly for hospitalisation DAMA by location, and odds ratios were not estimated.

Figures S6 and S7 show substantial differences over time in the percentage of hospital admissions and ED presentations ending in DAMA by hospital location.

### 3.4 30‐day readmissions/ re‐presentations

Of the 43,149 hospitalisations in this study, 6803 (15.8%) were followed by another hospitalisation within 30 days (Table 4). Compared to children for who the current admission was not recorded as DAMA, there was no evidence of an effect on children recorded as having a DAMA for 30‐day readmission (aOR 1.20, 95% CI 0.95, 1.51). The remaining characteristics and 30‐day hospital readmission are described in Supplemental Table 3.

**TABLE 4:** Associations between hospitalisation DAMA and 30‐day hospitalisation readmission, 2002–2018.

Hospitalisation DAMA	All hospitalisations (*n* = 43,149)	No readmission (*n* = 36,346)	Readmission (*n* = 6803)	OR (95% CI)	aOR (95% CI)^a^
Yes	684	560 (81.9%)	124 (18.1%)	1.39 (1.11, 1.74)	1.20 (0.95, 1.51)
No	42,465	35,786 (84.3%)	6679 (15.7%)	1.00 (reference)	1.00 (reference)

^a^ Minimal sufficient adjustment set used in adjusted model (see Figure S3): APGAR 5, Any previous Hospitalisation DAMA, Area socio‐economic index, Birthweight, Gestational age at birth, Hospital location, Maternal age at birth, Potentially preventable hospitalisation, Parity, Remoteness Area, Sex, Year of admission, Admission status, Age at admission.

Of the 232,082 ED presentations in this study, 74,151 (32.0%) were followed by another ED presentation within 30 days (Table 5). There was limited evidence that DAMA was associated with an decreased likelihood of another presentation to the ED within 30 days. The remaining characteristics and 30‐day ED re‐presentation are described the in Supplemental Table 4.

**TABLE 5:** Associations between emergency department DAMA and 30‐day emergency department re‐presentation, 2002–2018.

Emergency Department DAMA	All presentations (*n* = 232,082)	No re‐presentation (*n* = 157,931)	Re‐presentation (*n* = 74,151)	OR (95% CI)	aOR (95% CI)^a^
Yes	10,918	7785 (71.3%)	3133 (28.7%)	0.90 (0.86, 0.94)	0.96 (0.92, 1.01)
No	221,164	150,146 (67.9%)	71,018 (32.1%)	1.00 (reference)	1.00 (reference)

^a^ Minimal sufficient adjustment set used in adjusted models (see Figure S4): APGAR 5, Any previous Emergency DAMA, Area socio‐economic index, Birthweight, Gestational age at birth, Hospital location, Maternal age at birth, Parity, Remoteness Area, Sex, Year of presentation, Age at admission, Triage code.

## 4 COMMENT

### 4.1 Principal findings

In this paper we investigated hospitalisation and ED presentation DAMA among Western Australian Aboriginal children born 2002–2013, with a follow‐up period up to <5 years of age (2002–2018). Overall, there was a decrease in hospital DAMA and an increase in ED DAMA between 2002 and 2018. We found a range of characteristics associated with DAMA, including admission status, triage code and hospital location. There was no evidence of hospital DAMA being associated with an increased likelihood of hospital readmission within 30 days and limited evidence of ED DAMA being associated with re‐presenting to an ED within 30 days.

### 4.2 Strengths of the study

The strengths of this study include adding to the limited evidence on child DAMA. Given that children <5 years are discharged by an adult and not themselves, by providing evidence on the factors that influence a child's DAMA, we provide insight into how families interact with health systems. As such, our study of DAMA sheds light on this distinct health system issue. Relatedly, there are fewer studies on ED DAMA than on hospital DAMA. Studies of hospital and ED DAMA also tend to focus on specific sub‐populations, such as the cohort using a specific hospital or patients with a particular disease.^7^, ^8^, ^29^ As such, our population‐based study of children in both hospital and ED is an important addition to the literature. Our study also brings an explicit causal model, which provides a basis for future researchers to challenge and refine.

### 4.3 Limitations of the data

Our study had some limitations. While we have described a causal model, we have made some simplifications, such as excluding maternal DAMA. We were limited by the available data. For example, we did not have measures of constructs like cultural security, which has been shown to have a substantial impact on Aboriginal people's engagement and trust in health services. Nor do we have recorded waiting times which could impact the effect of triage status on DAMA. Finally, despite important differences in DAMA between metropolitan and regional and remote hospitals, small cell counts limited our ability to stratify our analyses of hospital DAMA by location.

### 4.4 Interpretation

For DAMA occurring in hospital between 2014 and 2018, the adjusted odds decreased by 75% compared to the period between 2002 and 2005. This reduction in hospital DAMA is potentially driven by improvements in regional and remote hospitals. During 2002–2018, hospitalisations in regional and remote hospitals were almost seven times more likely to DAMA than metropolitan hospitalisations. However, by 2018 the gap between metropolitan and regional and remote hospitals had substantially diminished. There has been a number of initiatives across WA Health that have been aimed at improving the care of Aboriginal people in hospital to reduce the event of a DAMA.^1^, ^11^, ^30^ It is likely that a culmination of these activities has reduced hospital DAMA in children.

Although hospital DAMA decreased, the adjusted odds of ED DAMA increased by 46% over the same period. The increase in ED DAMA over time is an unexpected finding, and although it appears there is peak in ED DAMA during 2007, there is still an increase in ED DAMA in 2014–2018 compared to 2002–2005. And while it is a favourable finding that children presenting to regional and remote EDs are less likely to DAMA than presentations in Perth metropolitan EDs, we also found a higher frequency of less urgent presentations compared with metropolitan Perth. Combined, these findings suggest that more primary healthcare services are required in these locations. It is well‐established that general practice (GP) access decreases with increasing remoteness in Australia, which is often reflected in increased emergency presentations for children in regional and remote and remote areas.^31^ For instance, it has been estimated that at least 20% of ED presentations in Western Australia could be managed in general practice.^32^

Admission status is an important predictor of whether or not a given hospitalisation will end with DAMA. For hospitalisations, we found a substantial association between admission status and DAMA, with an aOR for non‐elective admissions of more than six times greater than elective admissions from the waitlist. This finding is consistent with the extant literature. For example, in a study of children admitted to the hospitals in the Sydney Children's Hospitals Network, planned admissions were 31% less likely to DAMA than emergency admissions.^2^ Further, in a study of inpatient admission for ischaemic heart disease Katzenellenbogen et al.^7^ reported an aOR of 5.93 for emergency admissions compared with planned admissions.

We also found that ED presentations with a non‐urgent triage code resulted in 11.5 times greater adjusted odds of DAMA compared with presentations with an emergency triage code.^33^ This finding is consistent with descriptive studies of adult ED presentation DAMA in Italy, Saudi Arabia and Canada, with longer waiting times associated with an increased likelihood of DAMA.^29^, ^34^ A common theme in analyses of DAMA is dissatisfaction with waiting times.^3^, ^4^, ^35^, ^36^, ^37^ Another factor which drives the association between waiting times and DAMA is that some parents may choose to take their children home if symptoms improve. For example, in a study in a Canadian children's hospital, 37% of premature departures from Eds occurred after the child's symptoms resolved.^34^

## 5 CONCLUSIONS

In conclusion, our study highlights several factors associated with DAMA in hospitals and EDs, including hospital location, admission status, triage code and year of admission/ presentation. These findings have a number of practical implications. In particular, our results highlight the importance of admission status and triage code and suggest that further work is needed to reduce DAMA for admissions and presentations associated with less urgent entry to hospital and ED.

## AUTHOR CONTRIBUTIONS

NS and DMA conceived of the paper. BM obtained the data. DC and NS undertook the data analysis. All authors contributed to writing the paper.

## FUNDING INFORMATION

The Defying the Odds Study was funded by the Australian National Health and Medical Research Council (NHMRC) (grant no. 1078214).

## CONFLICT OF INTEREST STATEMENT

None.

## Supporting information


Figure S1

Figure S2

Figure S3

Figure S4

Figure S5

Figure S6

Figure S7

Table S1‐S4

## Data Availability

Data are available from the Western Australia Department of Health Data Linkage Branch with ethical approval through Western Australia Department of Health Human Ethics Committee (Migrated ID DOH‐201530). To maintain confidentiality and security, interested individuals may apply for access to linked data by contacting the Western Australian Data Linkage Branch. Contact details are dataservices@health.wa.gov.au; +61‐8‐9222 2370.
