# Supplementary figures and images for: Hospital and emergency department discharge against medical advice in Western Australian Aboriginal children aged 0–4 years from 2002 to 2018: A cohort study
Source: Paediatr Perinat Epidemiol. 2023 Nov 20;37(8):691–703. doi: 10.1111/ppe.13018 (PMC10946741; doi:10.1111/ppe.13018)

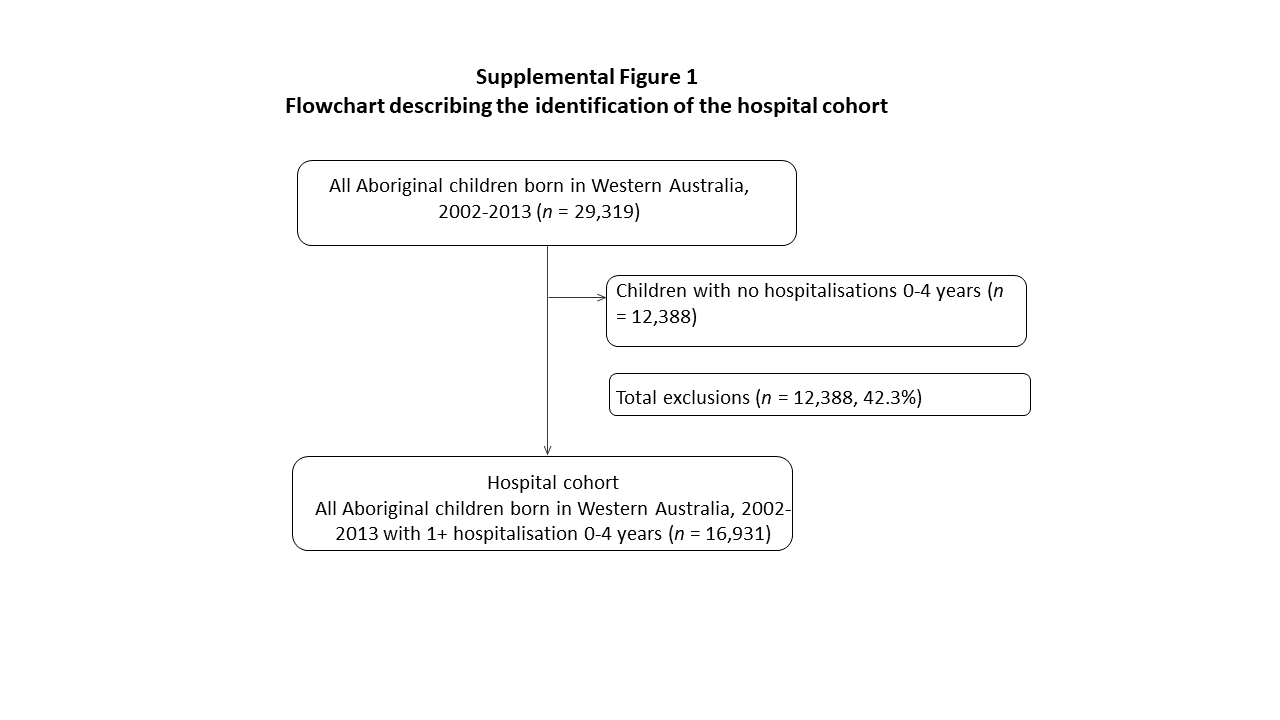

Supplement: Supplementary file 1 — Figure S1 [file PPE-37-691-s001.png]

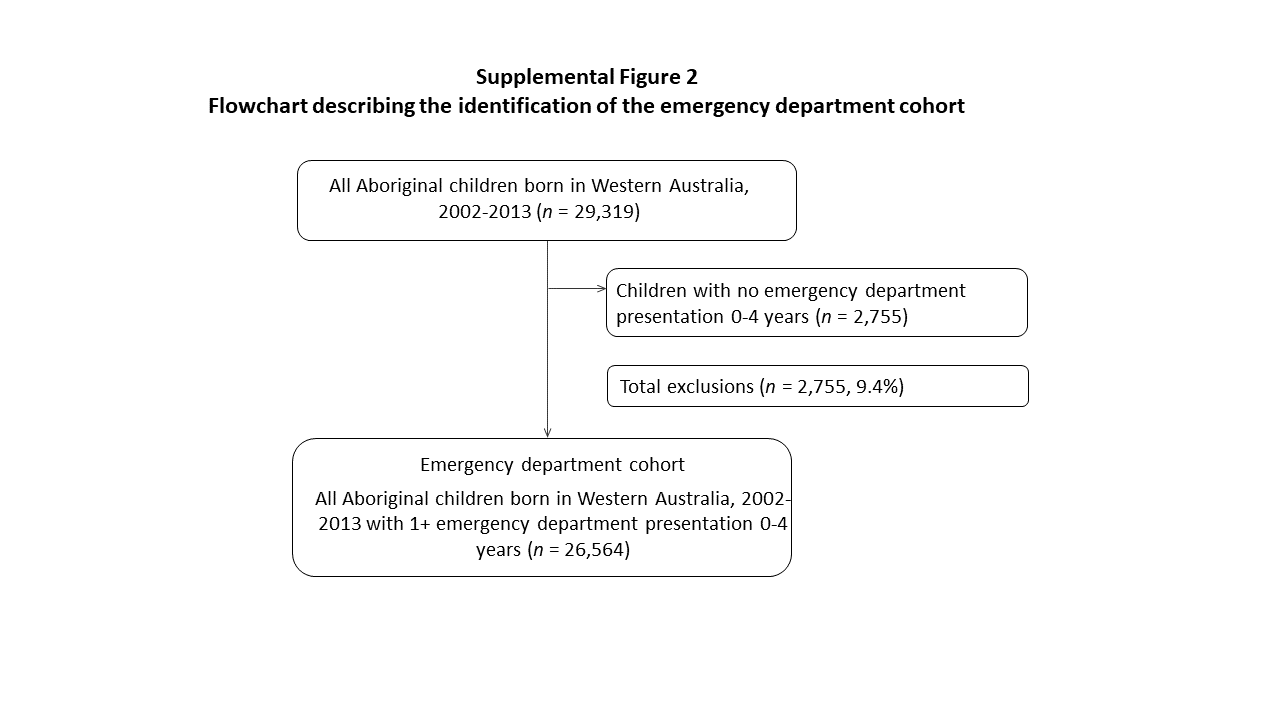

Supplement: Supplementary file 2 — Figure S2 [file PPE-37-691-s008.png]

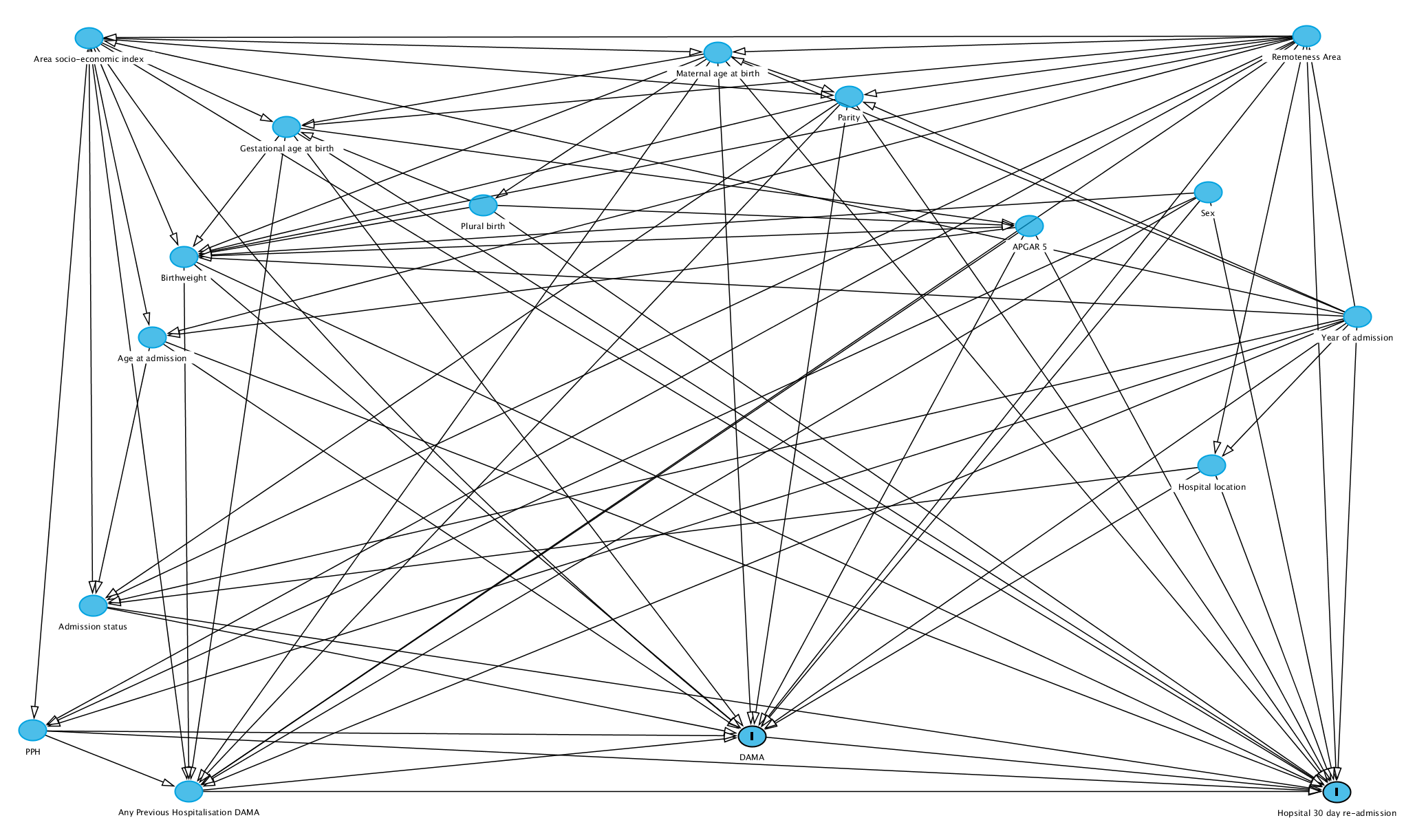

Supplement: Supplementary file 3 — Figure S3 [file PPE-37-691-s005.png]

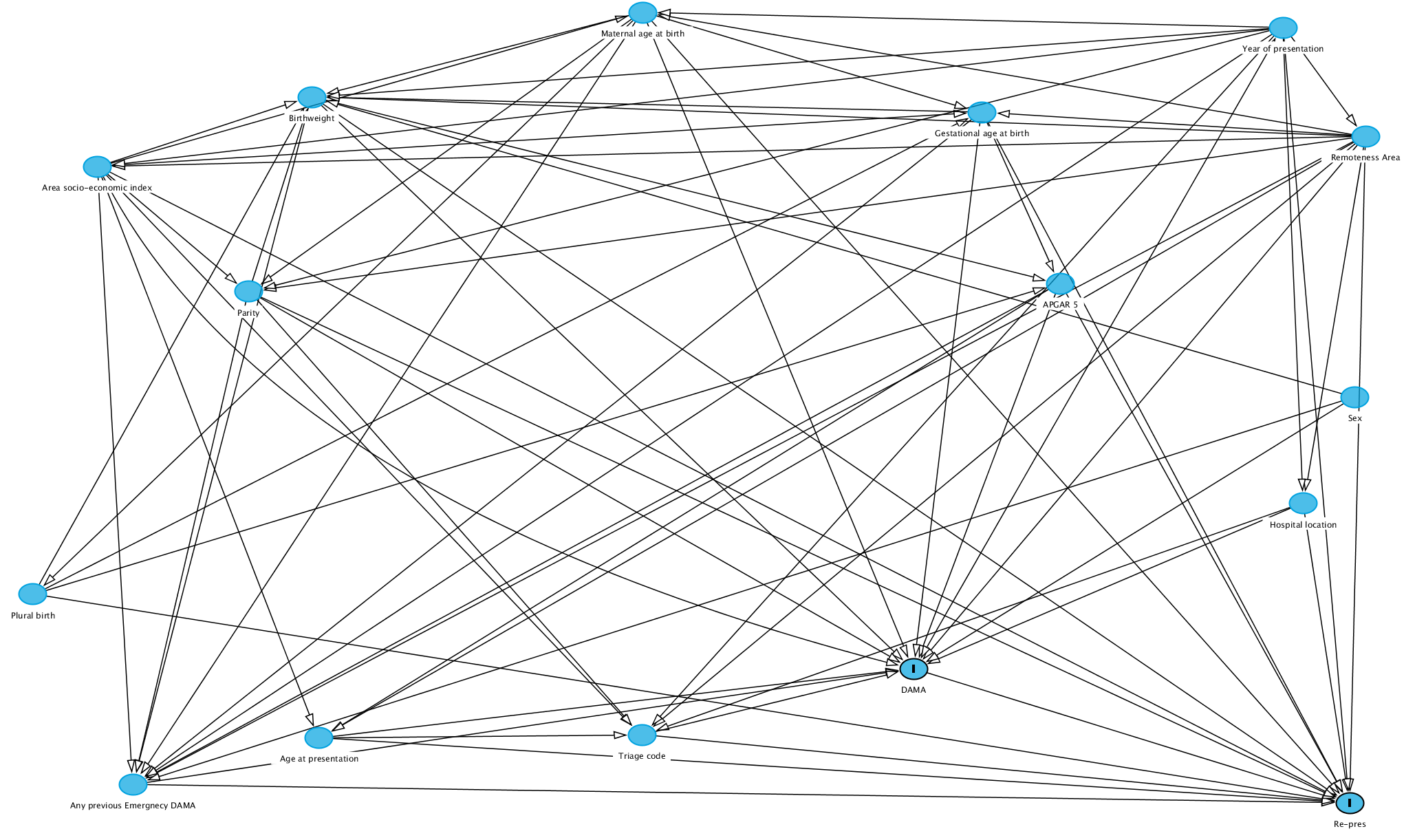

Supplement: Supplementary file 4 — Figure S4 [file PPE-37-691-s002.png]

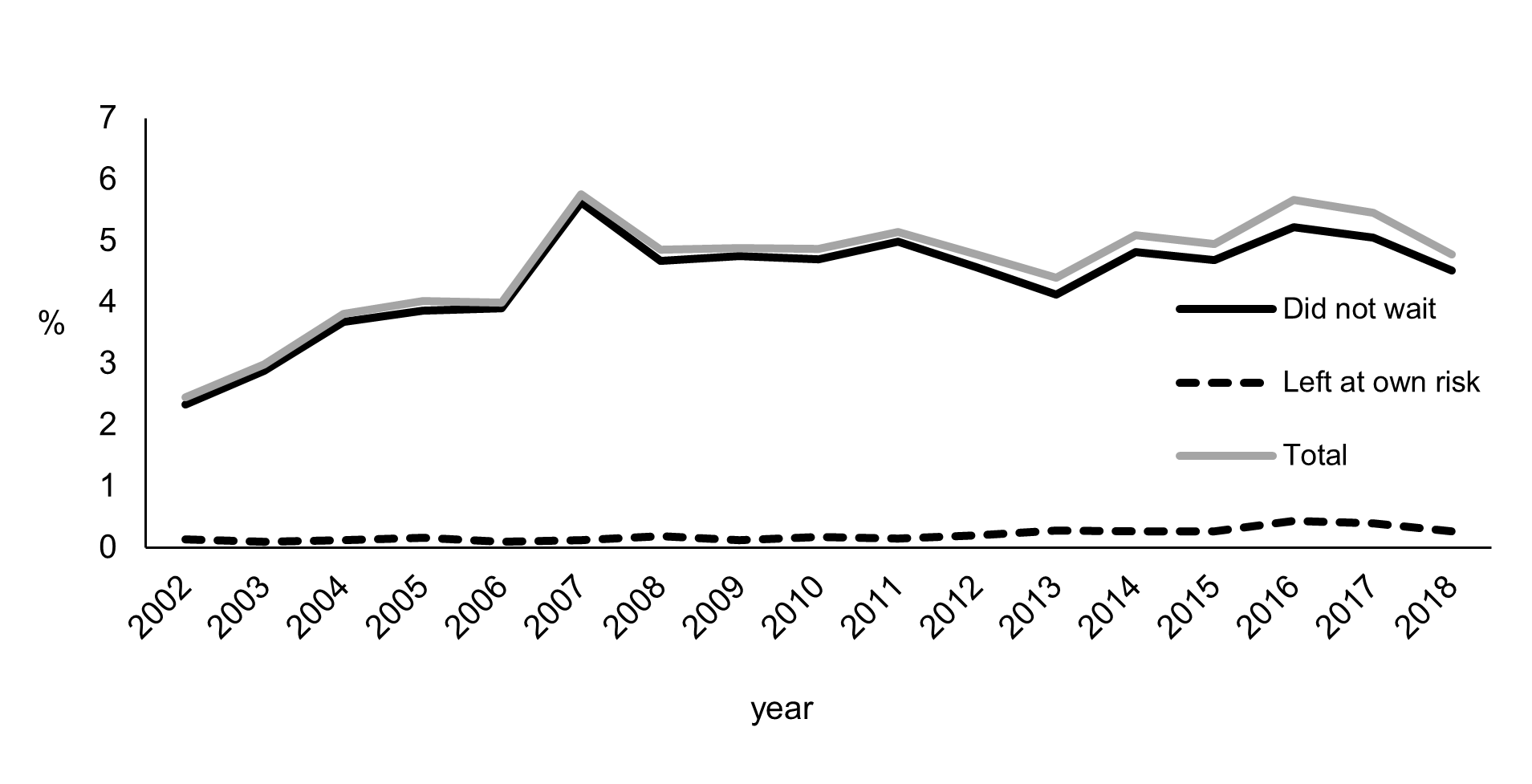

Supplement: Supplementary file 5 — Figure S5 [file PPE-37-691-s007.png]

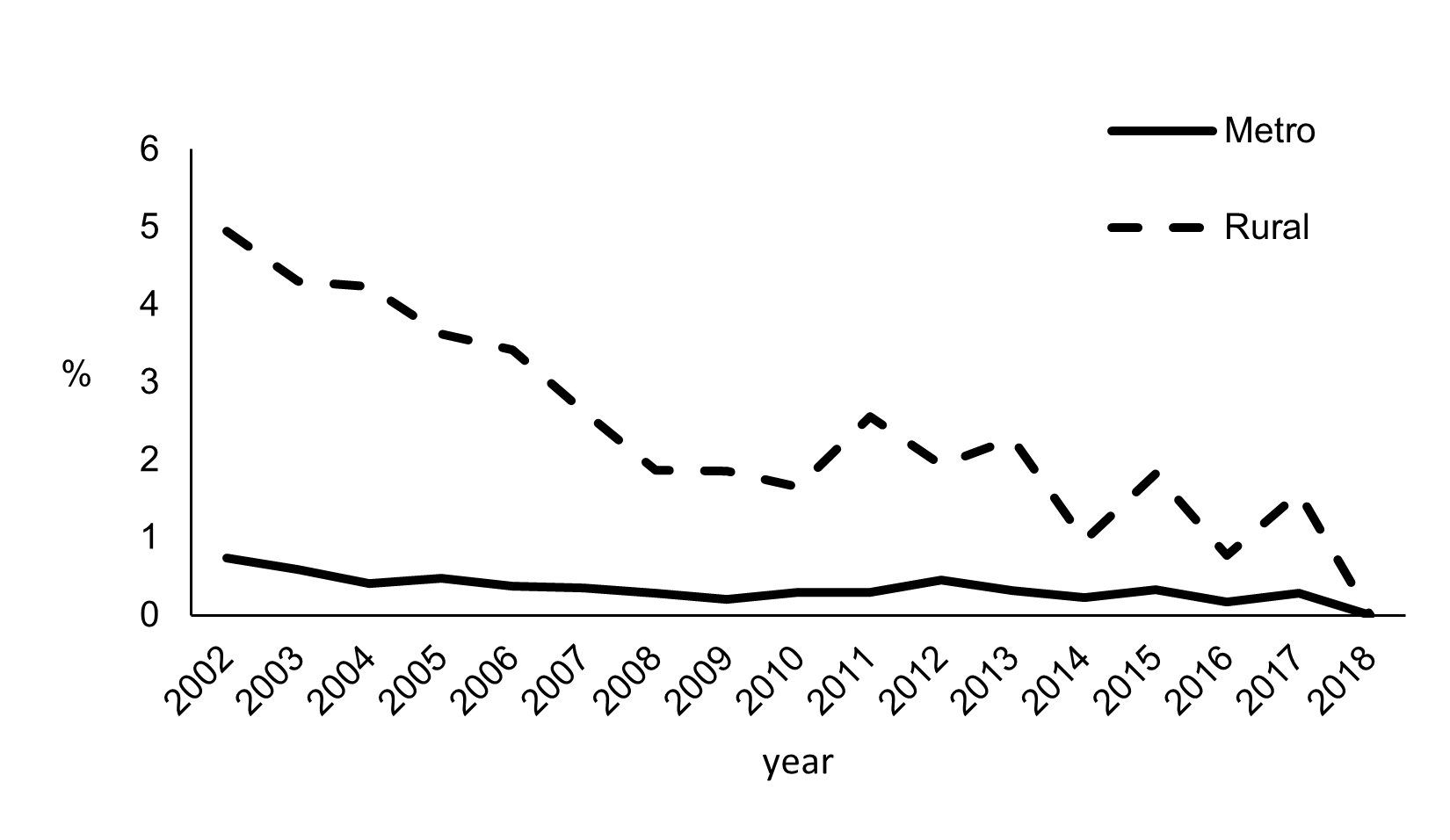

Supplement: Supplementary file 6 — Figure S6 [file PPE-37-691-s006.png]

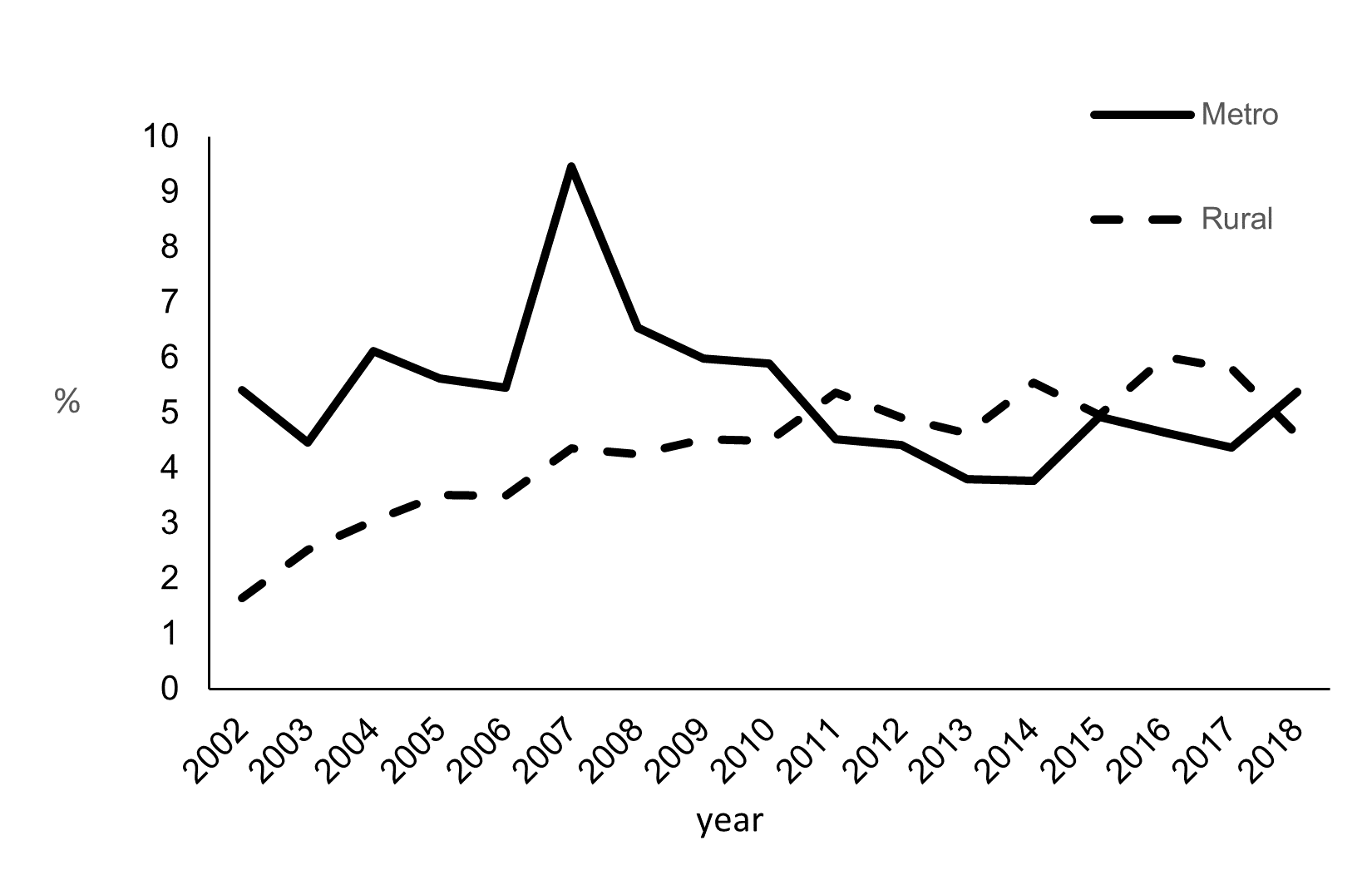

Supplement: Supplementary file 7 — Figure S7 [file PPE-37-691-s004.png]
