# Supplementary material for: Hospital and emergency department discharge against medical advice in Western Australian Aboriginal children aged 0–4 years from 2002 to 2018: A cohort study
Source: Paediatr Perinat Epidemiol. 2023 Nov 20;37(8):691–703. doi: 10.1111/ppe.13018 (PMC10946741; doi:10.1111/ppe.13018)
Supplement: Supplementary file 8 — Table S1‐S4 [file PPE-37-691-s003.docx]

**Supplemental Table 1. Description of characteristics of all hospital admissions up to age 5 by hospital location, 2002-2018**

| **Characteristic** | **All hospitalisations (n = 43,149)** | **Metropolitan hospitals (n = 18,693)** | **Regional and remote hospitals (n = 24,456)** |
| --- | --- | --- | --- |
| *Child* |  |  |  |
| Sex |  |  |  |
| Female | 18,799 | 7955 (42.3%) | 10,844 (57.7%) |
| Male | 24,350 | 10,738 (44.1%) | 13,612 (55.9%) |
| Plural birth |  |  |  |
| Yes | 1316 | 604 (45.9%) | 712 (54.1%) |
| No | 41,833 | 18,089 (43.2%) | 23,744 (56.8%) |
| Gestational age at birth |  |  |  |
| <37 weeks old | 8417 | 3820 (45.4%) | 4597 (54.6%) |
| ≥37 or more weeks | 34,732 | 14,873 (42.8%) | 19,859 (57.2%) |
| Birthweight |  |  |  |
| <2500g | 8034 | 3407 (42.4%) | 4627 (57.6%) |
| ≥2500g | 35,115 | 15,286 (43.5%) | 19,829 (56.5%) |
| APGAR 5 |  |  |  |
| <7 | 1373 | 582 (42.4%) | 791 (57.6%) |
| ≥7 | 41,679 | 18,073 (43.4%) | 23,606 (56.6%) |
| Missing | 97 | 38 (39.2%) | 59 (60.8%) |
| *Mother* |  |  |  |
| Parity |  |  |  |
| ≥3 previous births | 11,915 | 4725 (39.7%) | 7190 (60.3%) |
| <3 previous births | 31,234 | 13,968 (44.7%) | 17,266 (55.3%) |
| Maternal age at birth |  |  |  |
| <20 years old | 9522 | 3935 (41.3%) | 5587 (58.7%) |
| ≥20 years old | 33,627 | 14,758 (43.9%) | 18,869 (56.1%) |
| *Community* |  |  |  |
| Area socio-economic index |  |  |  |
| 1 (most disadvantaged) | 10,529 | 2351 (22.3%) | 8178 (77.7%) |
| 2 | 8020 | 4204 (52.4%) | 3816 (47.6%) |
| 3 | 8371 | 4078 (48.7%) | 4293 (51.3%) |
| 4 | 8436 | 4140 (49.1%) | 4296 (50.9%) |
| 5 (least disadvantaged) | 7744 | 3912 (50.5%) | 3832 (49.5%) |
| Missing | 49 | 8 (16.3%) | 41 (83.7%) |
| Remoteness Area |  |  |  |
| Major cities of Australia | 15,038 | 13,386 (89.0%) | 1652 (11.0%) |
| Regional Australia | 8581 | 2664 (31.0%) | 5917 (69.0%) |
| Remote Australia | 6033 | 1044 (17.3%) | 4989 (82.7%) |
| Very remote Australia | 13,448 | 1591 (11.8%) | 11,857 (88.2%) |
| missing | 49 | 8 (16.3%) | 41 (83.7%) |
| *Episode of service* |  |  |  |
| Any previous DAMA |  |  |  |
| Yes | 1443 | 202 (14.0%) | 1241 (86.0%) |
| No | 41,706 | 18,491 (44.3%) | 23,215 (55.7%) |
| Age at admission |  |  |  |
| Infant (<1) | 15,969 | 6,219 (38.9%) | 9750 (61.1%) |
| Other (1 < 5) | 27,180 | 12,474 (45.9%) | 14,706 (54.1%) |
| Year of admission |  |  |  |
| 2002-2005 | 8409 | 2996 (35.6%) | 5413 (64.4%) |
| 2006-2009 | 13,162 | 5649 (42.9%) | 7513 (57.1%) |
| 2010-2013 | 14,959 | 6748 (45.1%) | 8211 (54.9%) |
| 2014-2018 | 6619 | 3300 (49.9%) | 3319 (50.1%) |
| Potentially preventable hospitalisation |  |  |  |
| Yes | 23,657 | 8440 (35.7%) | 15,217 (64.3%) |
| No | 19,492 | 10,253 (52.6%) | 9239 (47.4%) |
| Admission status |  |  |  |
| Elective from waitlist | 6217 | 4986 (80.2%) | 1231 (19.8%) |
| Elective not from waitlist | 3737 | 1387 (37.1%) | 2350 (62.9%) |
| Emergency admission | 33,195 | 12,320 (37.1%) | 20,875 (62.9%) |

**Supplemental Table 2. Description of characteristics of all emergency department presentations up to age 5 years by hospital location, 2002-2018**

| **Characteristic** | **All presentations (n = 232,082)** | **Metropolitan hospitals (n = 60,335)** | **Regional and remote hospitals (n = 171,727)** |
| --- | --- | --- | --- |
| *Child* |  |  |  |
| Sex |  |  |  |
| Female | 107,990 | 27,669 (25.6%) | 80,321 (74.4%) |
| Male | 124,092 | 32,686 (26.3%) | 91,406 (73.7%) |
| Plural birth |  |  |  |
| Yes | 5238 | 1469 (28.0%) | 3769 (72.0%) |
| No | 226,844 | 58,886 (26.0%) | 167,958 (74.0%) |
| Gestational age at birth |  |  |  |
| <37 weeks old | 33,718 | 9537 (28.3%) | 24,181 (71.7%) |
| ≥37 or more weeks | 198,364 | 50,818 (25.6%) | 147,546 (74.4%) |
| Birthweight |  |  |  |
| <2500g | 30,980 | 8250 (26.6%) | 22,730 (73.4%) |
| ≥2500g | 201,102 | 52,105 (25.9%) | 14,8997 (74.1%) |
| APGAR 5 |  |  |  |
| <7 | 5140 | 1247 (24.3%) | 3893 (75.7%) |
| ≥7 | 226,526 | 59,039 (26.1%) | 167,487 (73.9%) |
| Missing | 416 | 69 (16.6%) | 347 (83.4%) |
| *Mother* |  |  |  |
| Parity |  |  |  |
| ≥3 previous births | 57,264 | 13,270 (23.2%) | 43,994 (76.8%) |
| <3 previous births | 174,818 | 47,085 (26.9%) | 127,733 (73.1%) |
| Maternal age at birth |  |  |  |
| <20 years old | 54,972 | 14,765 (26.9%) | 40,207 (73.1%) |
| ≥20 years old | 177,110 | 45,590 (25.7%) | 131,520 (74.3%) |
| *Community* |  |  |  |
| Area socio-economic index |  |  |  |
| 1 (most disadvantaged) | 60,440 | 5998 (9.9%) | 54,442 (90.1%) |
| 2 | 45,888 | 14,408 (31.4%) | 31,480 (68.6%) |
| 3 | 43,747 | 14,250 (32.6%) | 29,497 (67.4%) |
| 4 | 44,495 | 13,641 (30.7%) | 30,854 (69.3%) |
| 5 (least disadvantaged) | 37,355 | 12,045 (32.2%) | 25,310 (67.8%) |
| missing | 157 | 13 (8.3%) | 144 (91.7%) |
| Remoteness Area |  |  |  |
| Major cities of Australia | 65,405 | 49,879 (76.3%) | 15,526 (23.7%) |
| Regional Australia | 50,257 | 6169 (12.3%) | 44,088 (87.7%) |
| Remote Australia | 40,040 | 2007 (5.0%) | 38,033 (95.0%) |
| Very remote Australia | 76,223 | 2287 (3.0%) | 73,936 (97.0%) |
| Missing | 157 | 13 (8.3%) | 144 (91.7%) |
| *Episode of service* |  |  |  |
| Any previous DAMA |  |  |  |
| Yes | 50,001 | 10,374 (20.7%) | 39,627 (79.3%) |
| No | 182,081 | 49,981 (27.4%) | 132,100 (72.6%) |
| Age at admission |  |  |  |
| Infant (<1) | 73,152 | 20,431 (27.9%) | 52,721 (72.1%) |
| Other (1 < 5) | 158,930 | 39,924 (25.1%) | 119,006 (74.9%) |
| Year of presentation |  |  |  |
| 2002-2005 | 36,567 | 8780 (24.0%) | 27,787 (76.0%) |
| 2006-2009 | 76,692 | 19,962 (26.0%) | 56,730 (74.0%) |
| 2010-2013 | 80,261 | 21,860 (27.2%) | 58,401 (72.8%) |
| 2014-2018 | 38,562 | 9753 (25.3%) | 28,809 (74.7%) |
| Triage code |  |  |  |
| Resuscitation: immediate | 797 | 378 (47.4%) | 419 (52.6%) |
| Emergency: within 10 minutes | 3737 | 3147 (47.2%) | 3518 (52.8%) |
| Urgent: within 30 minutes | 51,655 | 18,095 (35.0%) | 33,560 (65.0%) |
| Semi-urgent: within 60 minutes | 120,810 | 36,875 (30.5%) | 83,935 (69.5%) |
| Non-urgent: within 120 minutes | 52,081 | 1832 (3.5%) | 50,249 (96.5%) |
| Missing | 74 | 28 (37.8%) | 46 (62.2%) |

**Supplemental Table 3. Associations between characteristics and 30-day hospitalisation readmission, 2002-2018.**

| **Characteristics** | **All hospitalisations (n = 43,149)** | **No re-admission (n = 36,346)** | **Re-admission (n = 6803)** | **aOR (95% CI)** | **aOR (95% CI)** | **Minimal sufficient adjustment set used in adjusted models (see Supplemental Figure 3)** |
| --- | --- | --- | --- | --- | --- | --- |
| *Child* |  |  |  |  |  |  |
| Sex |  |  |  |  |  | No adjustment is necessary to estimate the total effect of Sex on re-admission |
| Female | 18,799 | 16,071 (85.5%) | 2728 (14.5%) | 1.00 (reference) | 1.00 (reference) |  |
| Male | 24,350 | 20,275 (83.3%) | 4075 (16.7%) | 1.13 (1.05, 1.23) | 1.13 (1.05, 1.23) |  |
| Plural birth |  |  |  |  |  | Maternal age at birth |
| Yes | 1316 | 1082 (82.2%) | 234 (17.8%) | 1.44 (1.16, 1.8) | 1.45 (1.16, 1.8) |  |
| No | 41,833 | 35,264 (84.3%) | 6569 (15.7%) | 1.00 (reference) | 1.00 (reference) |  |
| Gestational age at birth |  |  |  |  |  | Area socio-economic index, Maternal age at birth, Plural birth, Remoteness Area |
| <37 weeks old | 8417 | 6708 (79.7%) | 1709 (20.3%) | 1.62 (1.47, 1.78) | 1.61 (1.46, 1.78) |  |
| ≥37 or more weeks | 34,732 | 29,638 (85.3%) | 5094 (14.7%) | 1.00 (reference) | 1.00 (reference) |  |
| Birthweight |  |  |  |  |  | Area socio-economic index, Gestational age at birth, Maternal age at birth, Parity, Plural birth, Remoteness Area, Sex, Year of admission |
| <2500g | 8034 | 6424 (80%) | 1610 (20%) | 1.65 (1.50, 1.82) | 1.33 (1.16, 1.52) |  |
| ≥2500g | 35,115 | 29,922 (85.2%) | 5193 (14.8%) | 1.00 (reference) | 1.00 (reference) |  |
| APGAR 5 |  |  |  |  |  | Birthweight, Gestational age at birth, Plural birth |
| <7 | 1373 | 1059 (77.1%) | 314 (22.9%) | 1.57 (1.25, 1.95) | 1.28 (1.02, 1.60) |  |
| ≥7 | 41,679 | 35,199 (84.5%) | 6480 (15.5%) | 1.00 (reference) | 1.00 (reference) |  |
| Missing | 97 | 88 (90.7%) | 9 (9.3%) |  |  |  |
| *Mother* |  |  |  |  |  |  |
| Parity |  |  |  |  |  | Area socio-economic index, Maternal age at birth, Remoteness Area, Year of admission |
| ≥3 previous births | 11,915 | 9782 (82.1%) | 2133 (17.9%) | 1.22 (1.12, 1.33) | 1.19 (1.08, 1.31) |  |
| <3 previous births | 31,234 | 26,564 (85%) | 4670 (15%) | 1.00 (reference) | 1.00 (reference) |  |
| Maternal age at birth |  |  |  |  |  | Area socio-economic index, Remoteness Area, Year of admission |
| <20 years old | 9522 | 8077 (84.8%) | 1445 (15.2%) | 1.03 (0.94, 1.13) | 0.99 (0.90, 1.08) |  |
| ≥20 years old | 33,627 | 28,269 (84.1%) | 5358 (15.9%) | 1.00 (reference) | 1.00 (reference) |  |
| *Community* |  |  |  |  |  |  |
| Area socio-economic index |  |  |  |  |  | Potentially preventable hospitalisation, Remoteness Area, Sex, Year of admission |
| 1 (most disadvantaged) | 10,529 | 8791 (83.5%) | 1738 (16.5%) | 1.24 (1.10, 1.4) | 1.00 (0.88, 1.14) |  |
| 2 | 8020 | 6763 (84.3%) | 1257 (15.7%) | 0.99 (0.87, 1.13) | 0.96 (0.84, 1.10) |  |
| 3 | 8371 | 7029 (84%) | 1342 (16%) | 1.08 (0.95, 1.23) | 1.03 (0.90, 1.18) |  |
| 4 | 8436 | 7195 (85.3%) | 1241 (14.7%) | 0.99 (0.87, 1.13) | 0.95 (0.84, 1.09) |  |
| 5 (least disadvantaged) | 7744 | 6526 (84.3%) | 1218 (15.7%) | 1.00 (reference) | 1.00 (reference) |  |
| Missing | 49 | 42 (85.7%) | 7 (14.3%) |  |  |  |
| Remoteness Area |  |  |  |  |  | Year of admission |
| Major cities of Australia | 15,038 | 12,835 (85.4%) | 2203 (14.6%) | 1.00 (reference) | 1.00 (reference) |  |
| Regional Australia | 8581 | 7327 (85.4%) | 1254 (14.6%) | 1.12 (1, 1.26) | 1.09 (0.97, 1.22) |  |
| Remote Australia | 6033 | 5238 (86.8%) | 795 (13.2%) | 1.18 (1.04, 1.34) | 1.17 (1.03, 1.33) |  |
| Very remote Australia | 13,448 | 10,904 (81.1%) | 2544 (18.9%) | 1.67 (1.51, 1.84) | 1.63 (1.47, 1.80) |  |
| Missing | 49 | 42 (85.7%) | 7 (14.3%) |  |  |  |
| *Episode of service* |  |  |  |  |  |  |
| Hospitalisation DAMA |  |  |  |  |  | APGAR 5, Any previous Hospitalisation DAMA, Area socio-economic index, Birthweight, Gestational age at birth, Hospital location, Maternal age at birth, Potentially preventable hospitalisation, Parity, Remoteness Area, Sex, Year of admission, Admission status, Age at admission |
| Yes | 684 | 560 (81.9%) | 124 (18.1%) | 1.39 (1.11, 1.74) | 1.20 (0.95, 1.51) |  |
| No | 42,465 | 35,786 (84.3%) | 6679 (15.7%) | 1.00 (reference) | 1.00 (reference) |  |
| Any previous Hospitalisation DAMA |  |  |  |  |  | APGAR 5, Area socio-economic index, Birthweight, Gestational age at birth, Maternal age at birth, Potentially preventable hospitalisation, Parity, Remoteness Area, Sex, Year of admission |
| Yes | 1443 | 1123 (77.8%) | 320 (22.2%) | 0.91 (0.76, 1.10) | 0.84 (0.69, 1.01) |  |
| No | 41,706 | 35,223 (84.5%) | 6483 (15.5%) | 1.00 (reference) | 1.00 (reference) |  |
| Age at admission (years) |  |  |  |  |  | APGAR 5, Area socio-economic index, Remoteness Area |
| Infant (<1) | 15,969 | 12,763 (79.9%) | 3206 (20.1%) | 2.18 (2.04, 2.33) | 2.17 (2.03, 2.31) |  |
| Other (1 < 5) | 27,180 | 23,583 (86.8%) | 3597 (13.2%) | 1.00 (reference) | 1.00 (reference) |  |
| Year of admission (years) |  |  |  |  |  | No adjustment is necessary to estimate the total effect of Year of admission on readmission |
| 2002-2005 | 8409 | 6772 (80.5%) | 1637 (19.5%) | 1.00 (reference) | 1.00 (reference) |  |
| 2006-2009 | 13,162 | 11,166 (84.8%) | 1996 (15.2%) | 0.64 (0.58, 0.70) | 0.64 (0.58, 0.70) |  |
| 2010-2013 | 14,959 | 12,654 (84.6%) | 2305 (15.4%) | 0.6 (0.54, 0.66) | 0.60 (0.54, 0.66) |  |
| 2014-2018 | 6619 | 5754 (86.9%) | 865 (13.1%) | 0.39 (0.34, 0.44) | 0.39 (0.34, 0.44) |  |
| Potentially preventable hospitalisation |  |  |  |  |  | Area socio-economic index, Remoteness Area, Sex, Year of admission |
| Yes | 23,657 | 20,532 (86.8%) | 3125 (13.2%) | 0.88 (0.82, 0.94) | 0.8 (0.79, 0.90) |  |
| No | 19,492 | 15,814 (81.1%) | 3678 (18.9%) | 1.00 (reference) | 1.00 (reference) |  |
| Admission status |  |  |  |  |  | Area socio-economic index, Hospital location, Parity, Remoteness Area, Year of admission, Age at admission |
| Elective from waitlist | 6217 | 5098 (82.0%) | 1119 (18.0%) | 1.00 (ref) | 1.00 (ref) |  |
| Elective not from waitlist | 3737 | 3026 (81.0%) | 711 (19.0%) | 1.46 (1.26, 1.68) | 1.17 (1.00, 1.36) |  |
| Emergency admission | 33,195 | 28,222 (85.0%) | 4973 (15.0%) | 1.64 (1.48, 1.82) | 1.30 (1.16, 1.45) |  |
| Hospital location |  |  |  |  |  | Remoteness Area, Year of admission |
| Perth metropolitan area | 18,693 | 15,669 (83.8%) | 3024 (16.2%) | 1.00 (ref) | 1.00 (ref) |  |
| Regional and remote | 24,456 | 20,677 (84.5%) | 3779 (15.5%) | 1.29 (1.20, 1.39) | 1.04 (0.95, 1.15) |  |

**Supplemental Table 4. Associations between characteristics and 30-day emergency department re-presentation, 2002-2018.**

| **Characteristics** | **All presentations (n = 232,082)** | **No re-presentation (n = 157,931)** | **Re-presentation (n = 74,151)** | **OR (95% CI)** | **aOR (95% CI)** | **Minimal sufficient adjustment set used in adjusted models (see Supplemental Figure 4)** |
| --- | --- | --- | --- | --- | --- | --- |
| *Child* |  |  |  |  |  |  |
| Sex |  |  |  |  |  | No adjustment is necessary to estimate the total effect of Sex on re-presentation |
| Female | 107,990 (100.0%) | 74,278 (68.8%) | 33,712 (31.2%) | 1.00 (reference) | 1.00 (reference) |  |
| Male | 124,092 | 83,653 (67.4%) | 40,439 (32.6%) | 1.08 (1.05, 1.11) | 1.08 (1.05, 1.11) |  |
| Plural birth |  |  |  |  |  | Maternal age at birth |
| Yes | 5238 | 3611 (68.9%) | 1627 (31.1%) | 0.96 (0.88, 1.05) | 0.97 (0.88, 1.06) |  |
| No | 226,844 | 154,320 (68%) | 72,524 (32%) | 1.00 (reference) | 1.00 (reference) |  |
| Gestational age at birth |  |  |  |  |  | Area socio-economic index, Maternal age at birth, Plural birth, Remoteness Area |
| <37 weeks old | 33,718 | 22,137 (65.7%) | 11,581 (34.3%) | 1.16 (1.12, 1.20) | 1.17 (1.13, 1.22) |  |
| ≥37 or more weeks | 198,364 | 135,794 (68.5%) | 62,570 (31.5%) | 1.00 (reference) | 1.00 (reference) |  |
| Birthweight |  |  |  |  |  | Area socio-economic index, Gestational age at birth, Maternal age at birth, Parity, Plural birth, Remoteness Area, Sex, Year of presentation |
| <2500g | 30,980 | 20,494 (66.2%) | 10,486 (33.8%) | 1.14 (1.10, 1.19) | 1.04 (0.99, 1.09) |  |
| ≥2500g | 201,102 | 137,437 (68.3%) | 63,665 (31.7%) | 1.00 (reference) | 1.00 (reference) |  |
| APGAR 5 |  |  |  |  |  | Birthweight, Gestational age at birth, Plural birth |
| <7 | 5140 | 3300 (64.2%) | 1840 (35.8%) | 1.18 (1.08, 1.29) | 1.13 (1.03, 1.24) |  |
| ≥7 | 226,526 | 154,318 (68.1%) | 72,208 (31.9%) | 1.00 (reference) | 1.00 (reference) |  |
| Missing | 416 | 313 (75.2%) | 103 (24.8%) |  |  |  |
| *Mother* |  |  |  |  |  |  |
| Parity |  |  |  |  |  | Area socio-economic index, Maternal age at birth, Remoteness Area, Year of presentation |
| ≥3 previous births | 57,264 | 38,233 (66.8%) | 19,031 (33.2%) | 1.04 (1.00, 1.07) | 1.04 (1.00, 1.07) |  |
| <3 previous births | 174,818 | 119,698 (68.5%) | 55,120 (31.5%) | 1.00 (reference) | 1.00 (reference) |  |
| Maternal age at birth |  |  |  |  |  | Area socio-economic index, Remoteness Area, Year of presentation |
| <20 years old | 54,972 | 37,232 (67.7%) | 17,740 (32.3%) | 1.08 (1.04, 1.11) | 1.04 (1.01, 1.07) |  |
| ≥20 years old | 177,110 | 120,699 (68.1%) | 56,411 (31.9%) | 1.00 (reference) | 1.00 (reference) |  |
| *Community* |  |  |  |  |  |  |
| Area socio-economic index |  |  |  |  |  | Remoteness Area, Year of presentation |
| 1 (most disadvantaged) | 60,440 | 38,499 (63.7%) | 21,941 (36.3%) | 1.37 (1.32, 1.43) | 1.16 (1.11, 1.21) |  |
| 2 | 45,888 | 31,533 (68.7%) | 14,355 (31.3%) | 1.14 (1.09, 1.19) | 1.12 (1.08, 1.18) |  |
| 3 | 43,747 | 30,154 (68.9%) | 13,593 (31.1%) | 1.1 (1.05, 1.15) | 1.08 (1.04, 1.13) |  |
| 4 | 44,495 | 30,771 (69.2%) | 13,724 (30.8%) | 1.12 (1.07, 1.17) | 1.09 (1.04, 1.14) |  |
| 5 (least disadvantaged) | 37,355 | 26,860 (71.9%) | 10,495 (28.1%) | 1.00 (reference) | 1.00 (reference) |  |
| Missing | 157 | 114 (72.6%) | 43 (27.4%) |  |  |  |
| Remoteness Area |  |  |  |  |  | Year of presentation |
| Major cities of Australia | 65,405 | 48,712 (74.5%) | 16,693 (25.5%) | 1.00 (reference) | 1.00 (reference) |  |
| Regional Australia | 50,257 | 35,659 (71%) | 14,598 (29%) | 1.18 (1.14, 1.23) | 1.18 (1.14, 1.23) |  |
| Remote Australia | 40,040 | 27,194 (67.9%) | 12,846 (32.1%) | 1.40 (1.34, 1.45) | 1.39 (1.34, 1.45) |  |
| Very remote Australia | 76,223 | 46,252 (60.7%) | 29,971 (39.3%) | 1.81 (1.75, 1.88) | 1.80 (1.73, 1.86) |  |
| Missing | 157 | 114 (72.6%) | 43 (27.4%) |  |  |  |
| *Episode of service* |  |  |  |  |  |  |
| DAMA |  |  |  |  |  | APGAR 5, Any previous Emergency DAMA, Area socio-economic index, Birthweight, Gestational age at birth, Hospital location, Maternal age at birth, Parity, Remoteness Area, Sex, Year of presentation, Age at admission, Triage code |
| Yes | 10,918 | 7785 (71.3%) | 3133 (28.7%) | 0.90 (0.86, 0.94) | 0.96 (0.92, 1.01) |  |
| No | 221,164 | 150,146 (67.9%) | 71,018 (32.1%) | 1.00 (reference) | 1.00 (reference) |  |
| Any previous Emergency DAMA |  |  |  |  |  | APGAR 5, Area socio-economic index, Birthweight, Gestational age at birth, Maternal age at birth, Parity, Remoteness Area, Sex, Year of presentation |
| Yes | 182,081 | 127,006 (69.8%) | 55,075 (30.2%) | 1.25 (1.21, 1.28) | 1.33 (1.29, 1.36) |  |
| No | 50,001 | 30,925 (61.8%) | 19,076 (38.2%) | 1.00 (reference) | 1.00 (reference) |  |
| Age at presentation (years) |  |  |  |  |  | APGAR 5, Area socio-economic index, Remoteness Area |
| Infant (<1) | 73,152 | 45,692 (62.5%) | 27,460 (37.5%) | 1.49 (1.46, 1.52) | 1.49 (1.46, 1.52) |  |
| Other (1 < 5) | 158,930 | 112,239 (70.6%) | 46,691 (29.4%) | 1.00 (reference) | 1.00 (reference) |  |
| Year of presentation (years) |  |  |  |  |  | No adjustment is necessary to estimate the total effect of Year of presentation on re-presentation |
| 2002-2005 | 36,567 | 23,730 (64.9%) | 12,837 (35.1%) | 1.00 (reference) | 1.00 (reference) |  |
| 2006-2009 | 76,692 | 51,635 (67.3%) | 25,057 (32.7%) | 0.90 (0.87, 0.92) | 0.90 (0.87, 0.92) |  |
| 2010-2013 | 80,261 | 55,136 (68.7%) | 25,125 (31.3%) | 0.84 (0.82, 0.86) | 0.84 (0.82, 0.86) |  |
| 2014-2018 | 38,562 | 27,430 (71.1%) | 11,132 (28.9%) | 0.75 (0.73, 0.77) | 0.75 (0.73, 0.77) |  |
| Triage code |  |  |  |  |  | Area socio-economic index, Hospital location, Parity, Remoteness Area, Year of presentation, Age at presentation |
| Resuscitation: immediate | 797 | 503 (63.1%) | 294 (36.9%) | 1.12 (0.95, 1.32) | 1.08 (0.92, 1.27) |  |
| Emergency: within 10 minutes | 6665 | 4420 (66.3%) | 2245 (33.7%) | 1.00 (ref) | 1.00 (ref) |  |
| Urgent: within 30 minutes | 51,655 | 35,429 (68.6%) | 16,226 (31.4%) | 0.86 (0.81, 0.91) | 0.83 (0.79, 0.88) |  |
| Semi-urgent: within 60 minutes | 120,810 | 85,001 (70.4%) | 35,809 (29.6%) | 0.77 (0.73, 0.82) | 0.75 (0.71, 0.8) |  |
| Non-urgent: within 120 minutes | 52,081 | 32,546 (62.5%) | 19,535 (37.5%) | 0.97 (0.91, 1.02) | 0.87 (0.82, 0.92) |  |
| Missing | 416 | 313 (75.2%) | 103 (24.8%) |  |  |  |
| Hospital location |  |  |  |  |  | Remoteness Area, Year of presentation |
| Perth metropolitan area | 60,355 | 44,790 (74.2%) | 15,565 (25.8%) | 1.00 (ref) | 1.00 (ref) |  |
| Regional and remote | 171,727 | 11,3141 (65.9%) | 58,586 (34.1%) | 1.49 (1.46, 1.52) | 1.04 (1.00, 1.08) |  |

**Supplemental Figure 1. Flowchart describing the identification of the hospital cohort.**

**Supplemental Figure 2. Emergency department cohort study flow diagram.**

**Supplemental Figure 3. Directed Acyclic Graph of Hospital DAMA and 30-day readmission^ab^.**

^a^ Outcomes marked with a ‘I’ symbol; ^b^ pph = potentially preventable hospitalisation

**Supplemental Figure 4. Directed Acyclic Graph of Emergency Department DAMA and 30-day re-presentation^a^.**

^a^ Outcomes marked with a ‘I’ symbol

**Supplemental Figure 5. Percent of emergency department presentations ending in DAMA by type of DAMA, 2002-2018.**

**Supplemental Figure 6. Percent of hospital admissions ending in DAMA by hospital location, 2002-2018.**

**Supplemental Figure 7. Percent of emergency department presentations ending in DAMA by hospital location, 2002-2018.**
